# Thermal Bending Simulation and Experimental Study of 3D Ultra-Thin Glass Components for Smartwatches

**DOI:** 10.3390/mi15101264

**Published:** 2024-10-17

**Authors:** Shunchang Hu, Peiyan Sun, Zhen Zhang, Guojun Zhang, Wuyi Ming

**Affiliations:** 1 Henan Key Laboratory of Intelligent Manufacturing of Mechanical Equipment, Zhengzhou University of Light Industry, Zhengzhou 450002, China; hushunchang2022@gmail.com (S.H.); peiyansun252@163.com (P.S.); 2 Guangdong Provincial Key Laboratory of Digital Manufacturing Equipment, Guangdong HUST Industrial Technology Research Institute, Dongguan 523808, China; zzhen@hust.edu.cn (Z.Z.); guojun_zhang_stu@163.com (G.Z.); 3 School of Aerospace Engineering, Huazhong University of Science and Technology, Wuhan 430074, China

**Keywords:** 3D ultra-thin glass components, simulation model, heat conduction, thermal bending forming, heating strategies

## Abstract

The heating system is an essential component of the glass molding process. It is responsible for heating the glass to an appropriate temperature, allowing it to soften and be easily molded. However, the energy consumption of the heating system becomes particularly significant in large-scale production. This study utilized G-11 glass for the simulation analysis and developed a finite element model for the thermal conduction of a 3D ultra-thin glass molding system, as well as a thermal bending model for smartwatches. Using finite element software, the heat transfer between the mold and the glass was modeled, and the temperature distribution and thermal stress under various processing conditions were predicted. The findings of the simulation, when subjected to a numerical analysis, showed that heating rate techniques significantly affect energy consumption. This study devised a total of four heating strategies. Upon comparison, optimizing with heating strategy 4, which applies an initial heating rate of 35 mJ/(mm^2^·s) during the initial phase (0 to 60 s) and subsequently escalates to 45 mJ/(mm^2^·s) during the second phase (60 to 160 s), resulted in a reduction of 4.396% in the system’s thermal output and a notable decrease of 7.875% in the heating duration, respectively. Furthermore, a single-factor research method was employed to study the forming process parameters. By comparing the numerical simulation results, it was found that within the temperature range of 615–625 °C, a molding pressure of 25–35 MPa, a heating rate of 1.5–2.5 °C/s, a cooling rate of 0.5–1 °C/s, and a pulse pressure of 45–55 Hz, the influence on residual stress and shape deviation in the glass was minimal. The relative error range was within the 20% acceptable limit, according to the experimental validation, which offered crucial direction and ideas for process development.

## 1. Introduction

As a crucial component of wearable devices, smartwatches merge the functionalities of traditional timepieces with those of smartphones, enabling the display and processing of various types of information [1,2,3]. In the design of smartwatches, 3D curved glass has become an essential material due to its distinctive optical properties, lightweight transparency, hardness, scratch resistance, and excellent weatherability [4,5,6]. As an amorphous material, glass requires molding within a specific temperature range to ensure adequate fluidity and plasticity [7,8]. Consequently, the heating system is an indispensable part of the glass molding process (GMP) [9,10,11]. It is responsible for heating the glass to an appropriate temperature, allowing it to soften and be easily molded. However, the energy consumption of the heating system becomes particularly prominent in large-scale production [12,13]. The heat conduction model must consider multiple factors, including the thermal conductivity of the mold and glass materials, contact thermal resistance, heating rate, and temperature differences between the mold and glass [14]. These factors influence the rate and efficiency of heat transfer, thereby affecting the quality of glass forming and the processing cycle [15].

Extensive research has been undertaken by numerous scholars on the topic of glass molding [16,17,18]. For instance, Su et al. [19] produced better geometric designs for desired lenses by simulating and predicting changes in the group refractive index using finite element techniques. The ultimate optical performance of molded glass components may be predicted through the use of compensating procedures aided by finite elements. In order to confirm that the material characteristics play a crucial role in predicting the amount of residual stresses in molded glass, Tao et al. [20] investigated the effects of the thermal expansion coefficient and specific heat capacity on the prediction of residual stresses in molded glass. Based on glass’s nonlinear thermal expansion characteristics, Yan et al. [21] suggested a two-step pressing procedure. By taking into account how specific heat and thermal conductivity depend on temperature, the phenomena of heat transmission was simulated. The shortest heating time and pressure changes were successfully predicted using a numerical model and observed glass characteristics.

Deeper exploration of the innate connection between process parameters and processing quality, as well as its optimization techniques, has been the main focus of recent studies [22,23,24]. For instance, the sustainability of ultra-thin GMP was investigated by Zhang et al. [25], who also showed how various process parameters affected energy efficiency. By utilizing both numerical modeling and experimental methodologies, He et al. [26] investigated the complete multiposition bending process of curved glass displays for smartphones. The principal parameters impacting residual stress and shape deviation in the finished goods were determined by analyzing the distribution of high-stress events. The GMP apparatus as a whole and the equipment implementation were carefully compared by Ming et al. [27], who also gave a complete description of the most current theoretical advancements in the use of high-frequency microwave and ultrasonic-assisted technologies in GMP.

Based on the current state of research and theoretical foundations, numerous factors significantly influence the quality of curved glass molding. The primary challenges of this study are as follows:

(1) Compared to large automotive dashboard glass [28] and curved smartphone glass [29], the 3D curved glass of smartwatches poses a significant challenge due to its ultra-thin thickness and small size, which greatly increases the difficulty of processing. Additionally, imprecise temperature control can easily lead to damage and quality issues during the GMP.
(2) To enhance production efficiency and ensure product quality, this study employed a dual-cavity mold design. Although this design can significantly boost production efficiency, it still faces considerable challenges in maintaining consistent quality for both the upper- and lower-molded glass components.

To investigate and optimize the energy consumption of the GMP, a finite element model of heat transfer was established for the molding die, conductive plate, and heating plate used for 3D ultra-thin glass components in smartwatches. Simulation analysis was conducted using MSC. Marc 2020 finite element software to model the heat transfer process between the mold and the glass, and to predict the temperature distribution and thermal stress of the mold under different processing conditions. By optimizing the heating system’s temperature rise strategy, energy utilization efficiency can be improved, promoting the sustainable development of GMP and reducing production costs in glass thermal-pressing processes. Additionally, this study developed a thermal bending model for the glass forming process of smartwatches. By conducting single-factor experiments, the forming process parameters were thoroughly investigated. This approach not only aided in optimizing the production process and reducing energy consumption but also enhanced the overall quality of the glass components. The results, validated through experiments, were found to be within an acceptable range, offering critical guidance and improvement suggestions for practical production processes.

## 2. Simulation Model

### 2.1. Heat Conduction Model

The generalized Maxwell model is a mechanical model used to describe the viscoelastic behavior of materials. It is an extension of the standard Maxwell model and is constructed by connecting multiple Maxwell units (combinations of springs and dashpots in series) in parallel. This allows for a more accurate representation of a material’s mechanical response over different timescales. In the generalized Maxwell model, each Maxwell unit has a distinct relaxation time, which enables the description of a material’s viscoelastic behavior under different loading frequencies or rates. By adjusting the parameters of each unit, the model can be fitted to experimental data to quantitatively describe the material’s mechanical properties, such as creep, relaxation, and dynamic modulus under various conditions [30]. This ability to describe behavior across multiple timescales makes the generalized Maxwell model particularly suitable for accurately simulating the complex behavior of glass during the molding process, including deformation, flow, and stress distribution. Therefore, this study adopts the generalized Maxwell model to investigate the GMP processing mechanism.

Various structural relaxation models can be used to simulate and investigate the structural relaxation phenomena of glass materials. Among these models, the method proposed by A.Q. Tool [31] is particularly noteworthy. It introduces the concept of a virtual temperature as an effective means of measuring the structural state of glass. Additionally, Tool’s approach expresses viscosity as a function of both the actual temperature and this virtual temperature, as detailed in Equation (1).

(1) $$\frac{dT_{f}}{dt}=\frac{T-T_{f}}{\tau_{p}}$$

In the equation, *τ*_p_ represents the structural relaxation time, which can be calculated using Equation (2).

(2) $$\tau_{p}=\tau_{0}\exp[-A_{1}T-A_{2}T_{f}]$$

In the equation, *τ*_0_, *A*_1_, and *A*_2_ are constants.

The number of additional parameters required to characterize the non-equilibrium glass structure is also a significant issue. The traditional Tool model uses a single virtual temperature parameter, *T*_f_, to describe the state of the glass. However, this approach has shown limitations in experimental observations. Narayanaswamy [32] introduced the concept of decay time and replaced the original relaxation function with the Kohlrausch extended exponential function (see Equation (3)). This modification, applied in the model expression (see Equation (4)), significantly enhanced the model’s ability to capture how temperature affects glass properties, resulting in more accurate and reliable predictions and interpretations.

(3) $$M_{v}\left(\xi \right)=\sum_{n}^{i=1}{\left(\omega_{g}\right)}_{i}\exp\left(-{\left(\frac{\xi }{\tau_{i}}\right)}^{b}\right)$$

(4) $$T_{f}\left(t\right)=T\left(t\right)-\int_{0}^{t}M_{v}\left(\xi \left(t\right)-\xi \left(t^{\prime }\right)\right)\frac{dT_{f}}{dt^{\prime }}\left(dt^{\prime }\right)$$

In the equation, *b* represents the Kohlrausch shape factor, while (*ω*_g_)*_i_* denotes the weight of the Prony exponent.

The two mechanisms of thermal transfer at the interface of glass materials include heat conduction within the object and heat convection with the external environment. In this study, it is assumed that the material under investigation exhibits isotropic properties, with physical characteristics such as density and thermal conductivity remaining constant during heat conduction. Based on these assumptions, the temperature distribution within the object can be described using the energy balance equation [33,34]. The temperature distribution curve within the object can be represented by Equation (5):

(5) $$\rho C_{p}\left(\frac{\partial T}{\partial t}\right)=k\left[\frac{1}{r}\frac{\partial }{\partial r}\left(r\frac{\partial T}{\partial r}\right)+\frac{1}{r^{2}}\frac{\partial^{2}T}{\partial \theta }+\frac{\partial^{2}T}{\partial z^{2}}\right]$$

In the equation, *ρ* represents the density of the object; *C*_p_ and *k* are the specific heat capacity and thermal conductivity, respectively; ∂*r*, ∂*θ*, and ∂*z* are the components in the radial, angular, and axial directions, respectively; and *T* denotes the temperature of the object itself.

Next, the heat convection and heat conduction at the object’s interface are also considered. Based on the principles of energy conservation and Fourier’s law of heat conduction, the transient temperature field *T*(*x*, *y*, *z*, *t*) of the object must satisfy the control equation, as given in Equation (6).

(6) $$\frac{\partial }{\partial x}(k_{x}\frac{\partial T}{\partial x})+\frac{\partial }{\partial y}(k_{y}\frac{\partial T}{\partial y})+\frac{\partial }{\partial z}(k_{z}\frac{\partial T}{\partial z})+\rho Q=\rho C_{T}\frac{\partial T}{\partial t}$$

In the equation, *Q*(*x*, *y*, *z*, *t*) represents the intensity of the heat source within the object, measured in watts per kilogram (W/kg).

**Figure 1:**
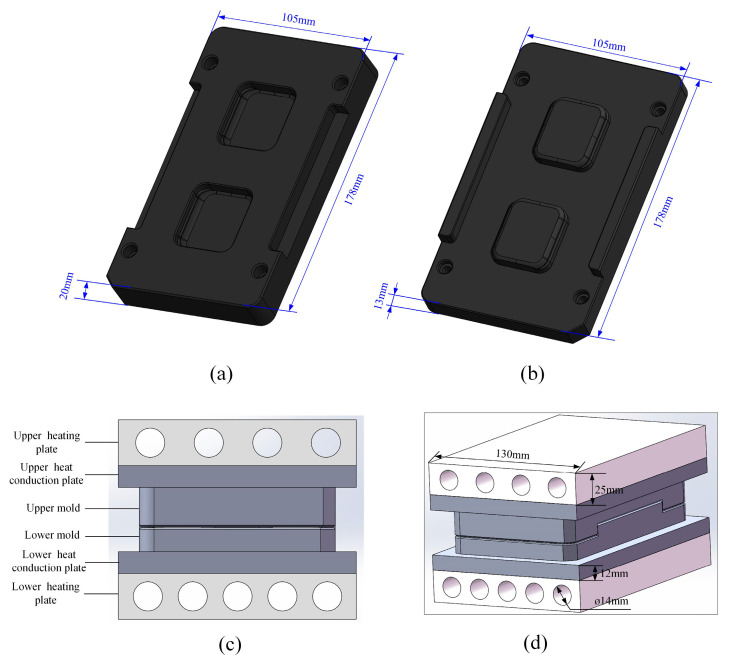
Dimensions of the molds and the heat transfer model, (**a**) upper mold dimensions, (**b**) lower mold dimensions, (**c**) positional relationship of heat transfer model, (**d**) heat transfer model dimensions.

A heat conduction model for the 3D ultra-thin glass components of smartwatches was developed. The spatial arrangement of the heating plate, heat conduction plate, and mold can be clearly understood, with the specific dimensions of the mold referenced in Figure 1a,b. The preformed dimensions of the 3D curved screen for the smartwatch were 40 × 35 × 0.3 mm, the dimensions of the heat conduction plate were set to 200 × 130 × 12 mm, while the heating plate was slightly thicker at 200 × 130 × 20 mm, as in Figure 1c,d. Notably, the upper heating plate was embedded with four heating tubes, and the lower heating plate contained five, both with a uniform bore diameter of 14 mm to ensure uniform and efficient heating. Assuming the heating plates could fully absorb the heat released by the heating tubes, heat flux density was used as a metric to avoid repeatedly establishing complex heating tube models. For material selection, WC was used for the heat conduction plate, SUS 310S for the heating plate, and the mold was made of graphite material to ensure optimal processing results. Graphite possesses excellent high-temperature resistance, electrical conductivity, thermal conductivity, and corrosion resistance [35]. Detailed mechanical and thermal performance parameters of WC, SUS 310S, and graphite are provided in Table 1, offering crucial reference data for the hot-pressing process of glass.

**Figure 2:**
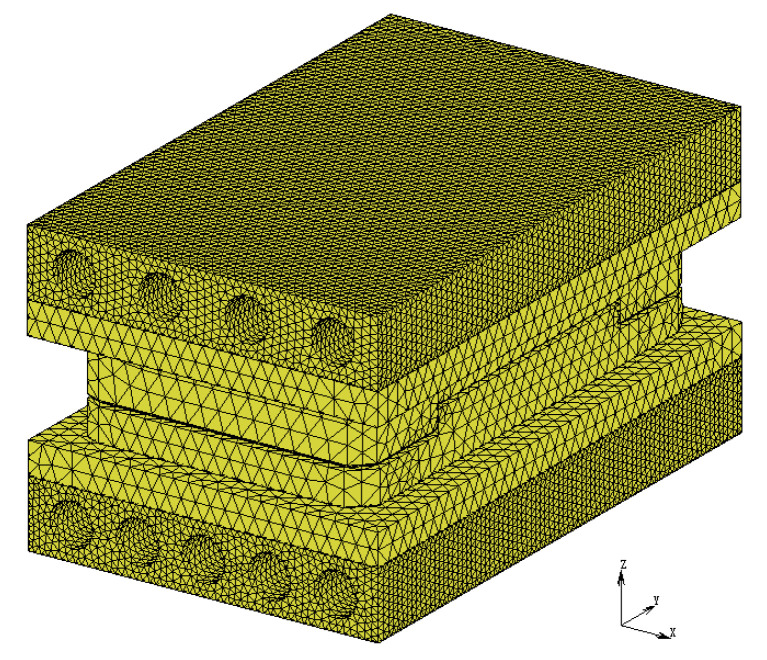
Three-dimensional finite element meshing of the GMP heat transfer model.

To accurately simulate the actual conditions, tetrahedral elements were used to mesh the heat conduction model of the 3D ultra-thin glass components for smartwatches. As shown in Figure 2, the meshing of the upper and lower molds resulted in a total of 50,090 elements. The meshing of the upper and lower heat conduction plates yielded 28,479 elements. Due to the presence of heating tubes, the heating plate was meshed into 358,854 elements. Additionally, to ensure the model’s accuracy, initial boundary conditions were defined based on the processing environment of the GMP, as detailed in Table 2.

### 2.2. Thermal Bending Forming Model

This study utilized G-11 glass for the simulation analysis, with the reference temperature set at 618 °C, reflecting its actual processing parameters. Table 3 presents the mechanical and thermal properties of graphite, which is used to fabricate molds. Graphite is commonly chosen for glass component molds due to its unique chemical and physical stability and its ability to perform reliably at high temperatures ranging from 800 to 1000 °C. In isothermal glass molding, the glass and mold are at the same temperature, which limits the glass temperature to protect the mold’s surface and extend its lifespan. Nonisothermal molding, however, allows different temperatures for the glass and mold, minimizing temperature variations during rapid molding. This method avoids prolonged heating and cooling cycles, maintaining a consistently low mold temperature, which reduces oxidation, wear, glass sticking, and thermal stress. These benefits extend the mold’s service life and lower the costs of precision mold manufacturing [36,37]. The relaxation modulus of glass exhibits a strong temperature dependency, characterized by slow relaxation at low temperatures and fast relaxation at high temperatures. Glass can retain its shape at various temperatures while shifting along the logarithmic time axis, which is a simple thermorheological behavior of glass described by the William–Landel–Ferry equation [38]. According to Narayanaswamy [32], based on A.Q. Tool’s model, structural relaxation in glass materials is described using relaxation time. During the heating phase, the viscosity variation of the glass was modeled using the William–Landel–Ferry equation. Furthermore, the Narayanaswamy model was applied to elucidate the intrinsic relationship between temperature and structural relaxation during the annealing and cooling stages [15,39]. Table 4 offers comprehensive details on the glass’s stress relaxation and structural properties [40,41].

## 3. Heat Conduction Simulation

### 3.1. Energy Calculation Model

**Figure 3:**
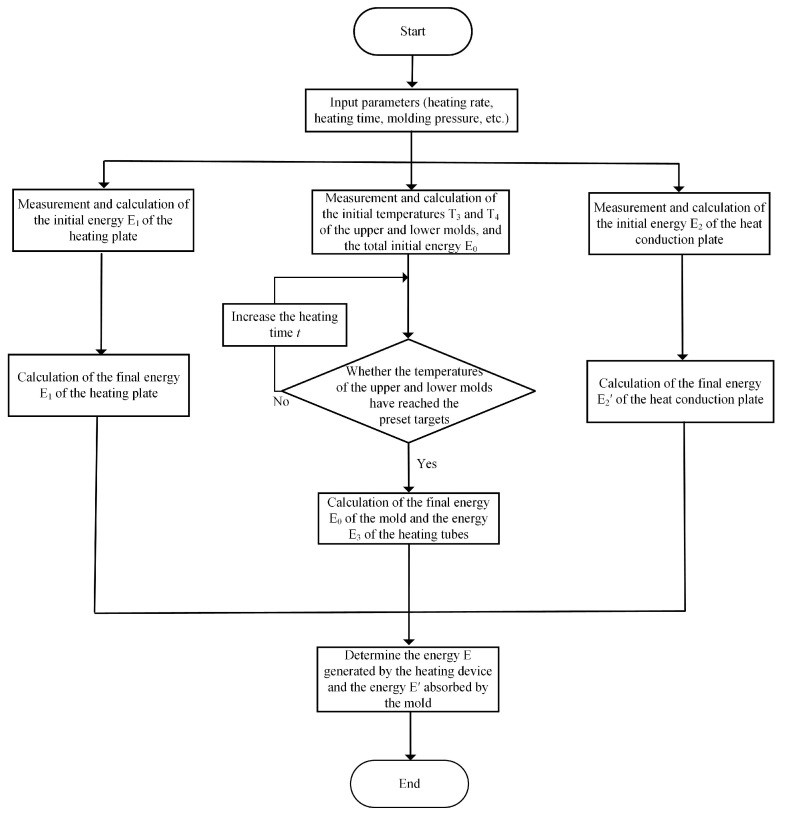
The energy consumption calculation process of the GMP system during heating.

The GMP system’s energy consumption estimation procedure during the heating phase is shown in Figure 3. The first step in this procedure was to calculate the heat that was transmitted to the mold through the heating plate and heat conduction plate. The remaining portion of the energy produced by the heating device was made up of the heat that was directly produced by the heating tubes. Equation (7) was used to determine the internal energy loss of the heating plate due to heat transfer (*E*_1_″) based on measurements of the starting temperature (*T*_1_) and the ending temperature (*T*_1_′).

(7) $$E_{1}^{\prime \prime }=E_{1}-E_{1}^{\prime }=m_{1}c_{1}(T_{1}-T_{1}^{\prime })$$

where *E* and *E*_1_′ represent the initial and final energy of the heating plate, respectively, and m and c denote the mass and specific heat capacity. Similarly, Equation (8) may be used to determine the heat spreader’s internal energy loss (*E*_2_″).

(8) $$E_{2}^{\prime \prime }=E_{2}-E_{2}^{\prime }=m_{2}c_{2}(T_{2}-T_{2}^{\prime })$$

Equation (9) determines the amount of heat produced by the heating tube.

(9) $$E_{3}=n{\bar{q}}_{f}St$$

where *S* is the heating tubes’ surface area (mm^2^), *n* is the number of heating tubes, and *t* is the heating duration (s). Equation (10) may thus be used to determine the energy produced by the heating equipment (*E*).

(10) $$E=E_{1}^{\prime \prime }+E_{2}^{\prime \prime }+E_{3}$$

Equation (11) may be used to describe the energy that the mold has absorbed.

(11) $$E^{\prime }=E_{0}^{\prime }-E_{0}=m_{3}c_{3}(T_{3}^{\prime }-T_{3})+m_{4}c_{4}(T_{4}^{\prime }-T_{4})$$

where *E*_0_ and *E*_0_′ represent the initial and final energy of the mold, respectively; *T*_3_ and *T*_4_ are the initial temperatures of the upper and lower molds, while *T*_3_′ and *T*_4_′ are the final temperatures of the upper and lower molds; *m*_3_ and *m*_4_ denote the mass of the upper and lower molds, respectively; and *c*_3_ is the specific heat capacity of the mold.

### 3.2. Mold Simulation Analysis

**Figure 4:**
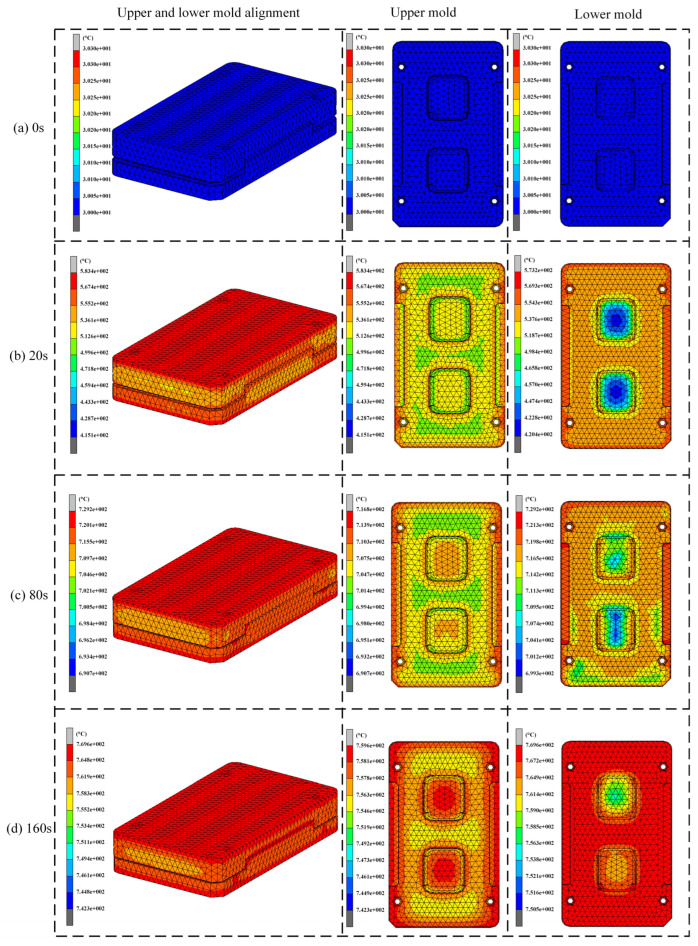
Temperature distribution of the mold during the heating stage (0.35 kW per heating tube; heating time: 0–160 s).

**Figure 5:**
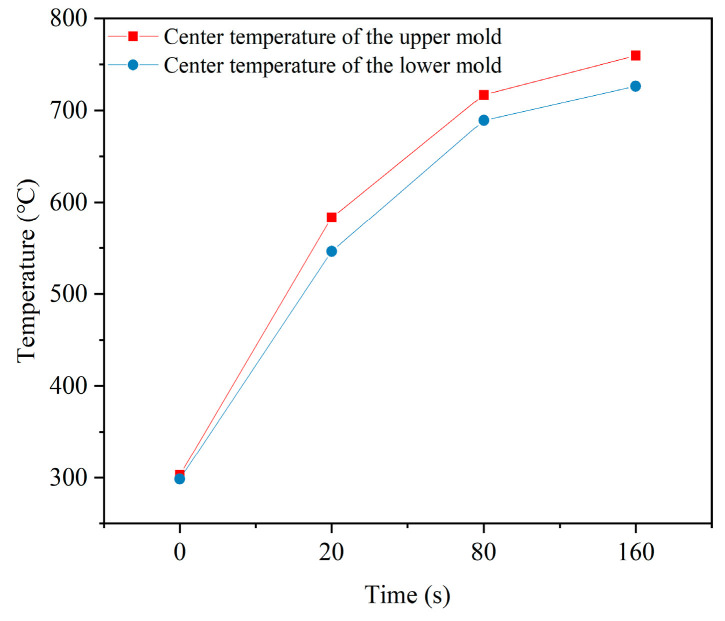
Center temperatures of the upper and lower molds (0.35 kW per heating tube; heating time: 160 s).

As shown in Figure 4, at the initial stage, the temperature of the mold was set to 30 °C, with the upper heating plate set to 800 °C and the lower heating plate to 810 °C, as shown in Figure 4a. After 20 s of heating, the heat conduction plate began the cooling phase. At this point, the temperature of the mold started to gradually rise, as shown in Figure 4b. It is notable that the temperature distribution within the molds is not uniform, with the upper mold reaching a maximum temperature of 583.4 °C and the lower mold reaching 573.2 °C. As heating time progressed, the temperature of the mold continued to rise, though the rate of heating gradually decreased, as shown in Figure 4c,d. The thermal loss effect became more pronounced at higher temperatures. Additionally, Figure 4d shows that the temperature at the end of the lower mold was higher than that of the upper mold. This was primarily due to the lower heating plate being equipped with more heating tubes and having a larger heated area, resulting in a faster heating rate for the lower mold. The temperature variation trends at the highest points of the upper and lower molds are shown in Figure 5.

**Figure 6:**
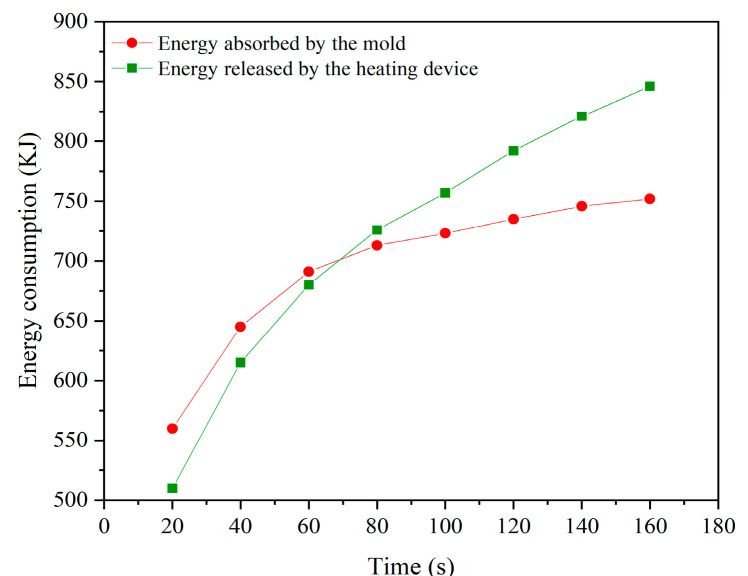
Energy consumption during the heating stage.

Figure 6 provides a detailed depiction of the energy consumption during the heating phase of the GMP system. Notably, during the initial 0–60 s, the thermal demand of the mold exceeds the heat supplied by the heating device, necessitating the absorption of additional heat from environmental sources such as high-temperature nitrogen. As heating continues, the mold temperature gradually increases due to the reduced temperature difference between the mold and the heating device, which results in a lower rate of heat transfer. Additionally, during the heating process, other heat transfer mechanisms such as convection and radiation become significant. As the mold temperature rises, the effects of convection and radiation intensify, potentially reducing the amount of heat absorbed from the heating device and thereby decreasing the efficiency of heat conduction. Combining the simulation data from Figure 4 and Figure 5, it is observed that both the maximum temperature rise and the temperature gradient of the mold decrease progressively as the heating process advances. However, some issues persist, particularly in the latter part of the heating phase, such as prolonged heating cycles leading to reduced energy efficiency and uneven heat distribution within the cavity. These issues adversely affect the heating process and decrease overall energy utilization efficiency.

### 3.3. Impact of Heating Rate on Production Energy Consumption

**Figure 7:**
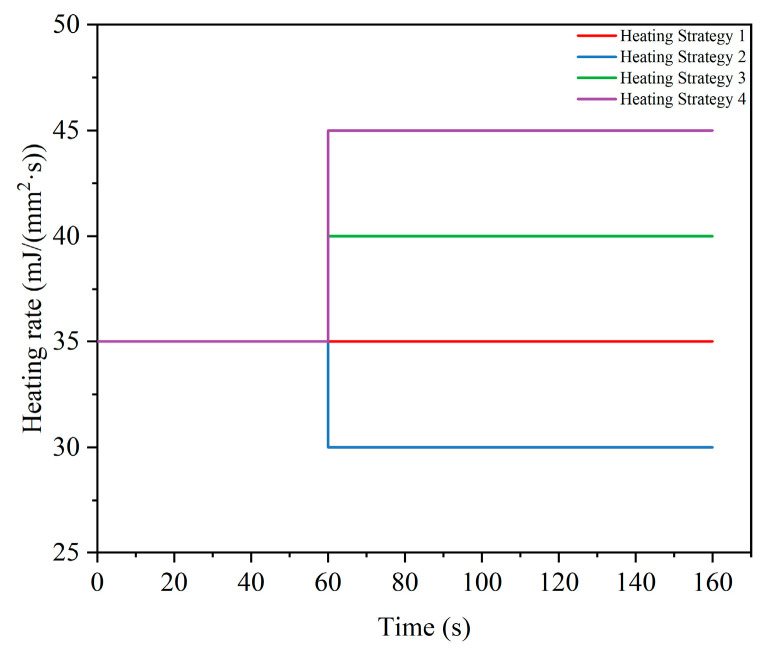
Diagram of different heating rate strategies.

This study employed four different heating rate strategies to simulate the mold heating process and to examine the precise impact of heating rate on energy consumption, as shown in Figure 7. Strategy 1 maintained a constant heating rate of 35 mJ/(mm^2^·s) throughout the heating phase (0 to 160 s), a value based on empirical data from experiments or previous studies, which ensured that the mold reached the target temperature within a reasonable time. Strategies 2, 3, and 4 adopted the same initial heating rate of 35 mJ/(mm^2^·s) during the initial phase (0 to 60 s) as strategy 1. However, during the second phase of heating (61 to 160 s), strategy 2 adjusted the heating rate to 30 mJ/(mm^2^·s). This strategy aimed to reduce energy consumption and potentially improve heating uniformity by decreasing the heating rate as the mold approached the target temperature. During the second phase (61 to 160 s), strategies 3 and 4 raised the heating rate to 40 mJ/(mm^2^·s) and 45 mJ/(mm^2^·s), respectively, to examine the impact of increased heating rates on energy consumption and heating efficiency.

**Figure 8:**
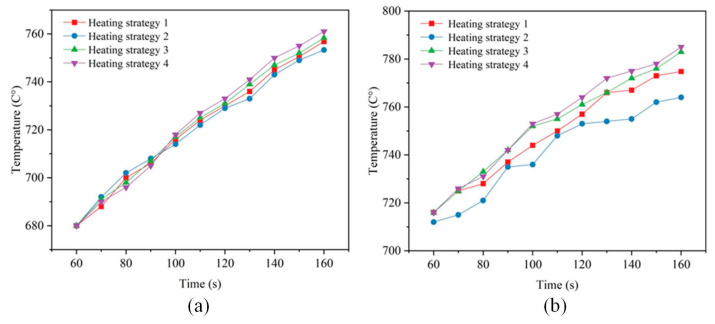
Center temperatures of the molds under different heating strategies, (**a**) upper mold, (**b**) lower mold.

Figure 8a illustrates that the temperature curves at the center of the upper mold under the four different heating rate strategies exhibited similar trends. As heating time progressed, temperature differences between the curves began to emerge, though the maximum temperature difference remained within 8 °C. Specifically, the center temperature of the upper mold varied slightly with different heating strategies, reaching 756.8 °C, 753.3 °C, 758.3 °C, and 761 °C, respectively. This indicates that variations in heating rate had a relatively limited impact on the temperature increase in the upper mold. In contrast, Figure 8b shows that the center temperature of the lower mold exhibited significant differences at the end of heating due to different heating strategies, ranging from 774.8 °C to 785.2 °C, with a maximum temperature difference of 21.5 °C. This indicates that changes in heating rate had a significant effect on the lower mold’s temperature rise. Although strategies 3 and 4 showed similar heating performances, their effects on the lower mold temperature still differed. Overall, the analysis concluded that the influence of the four different heating strategies on the lower mold was significantly greater than on the upper mold. This discrepancy was primarily due to the additional heating tube in the lower mold. The increased heating rate provided by the additional tube played a critical role in raising the temperature of the lower mold. Therefore, in practical applications, heating strategies should be tailored to the characteristics of the lower mold to achieve optimal heating performance.

### 3.4. Heat Conduction Simulation Modeling Results and Analysis

**Figure 9:**
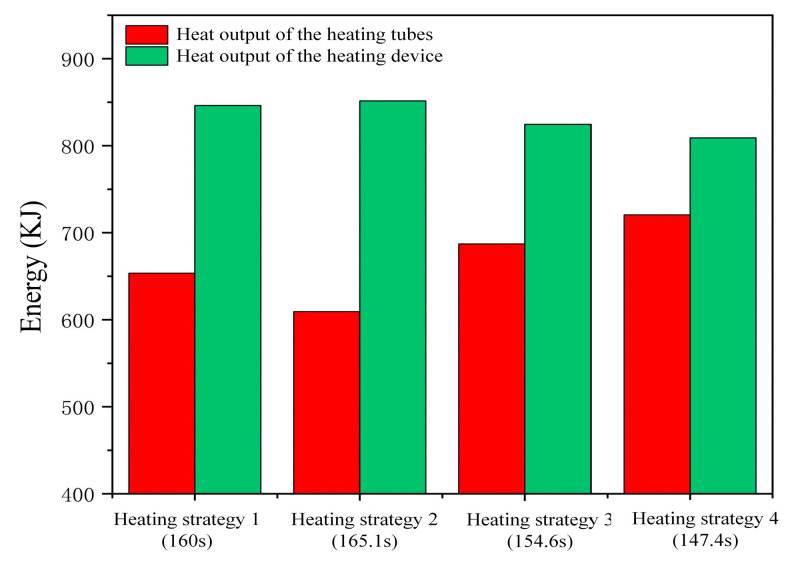
Heat output of the heating tubes and heating device under different heating rate strategies.

As shown in Figure 9, this investigation contrasted the heat output of the heating apparatus and tubes under four distinct ramp-up rate schemes. Experimental data revealed that the heat output from the heating tubes was 653.6 kJ, 609.4 kJ, 687.2 kJ, and 720.5 kJ, respectively, for the four strategies, while the heat output from the heating equipment was 846.3 kJ, 851.6 kJ, 824.7 kJ, and 809.1 kJ, respectively. It was observed that a lower heating rate in the second phase resulted in reduced heat output from the heating tubes, whereas a higher heating rate led to decreased heat output from the heating equipment. This is because higher heating rates achieve the target temperature more quickly, reducing heating time and energy consumption.

Compared with strategy 1, strategy 2 in the second phase resulted in a slight increase of 0.9993% in the heat output from the heating device, whereas strategy 4 reduced the heat output by 4.396% and shortened the heating time by 7.875%. However, excessively high heating rates may lead to increased internal stresses in the material and difficulties in controlling dimensional accuracy. Therefore, selecting the optimal ramp-up rate requires a balance between processing performance and energy consumption. Based on the evaluation, strategy 4 was deemed acceptable and held potential for reducing production energy consumption and shortening production cycles. Thus, this study favors the use of strategy 4 for optimization.

This study developed a thermal conduction finite element model for the 3D ultra-thin glass molding system used in smartwatches, including the mold, heat spreader, and heating plates. Simulations were performed using MSC. Marc 2020 finite element software to model the heat transfer between the mold and the glass and to predict temperature distribution and thermal stress under various processing conditions. The results indicate that optimizing with strategy 4 reduced the heat output and heating time of the system by 4.396% and 7.875%, respectively.

## 4. Thermal Bending Forming Simulation

### 4.1. Heating Process Simulation

**Figure 10:**
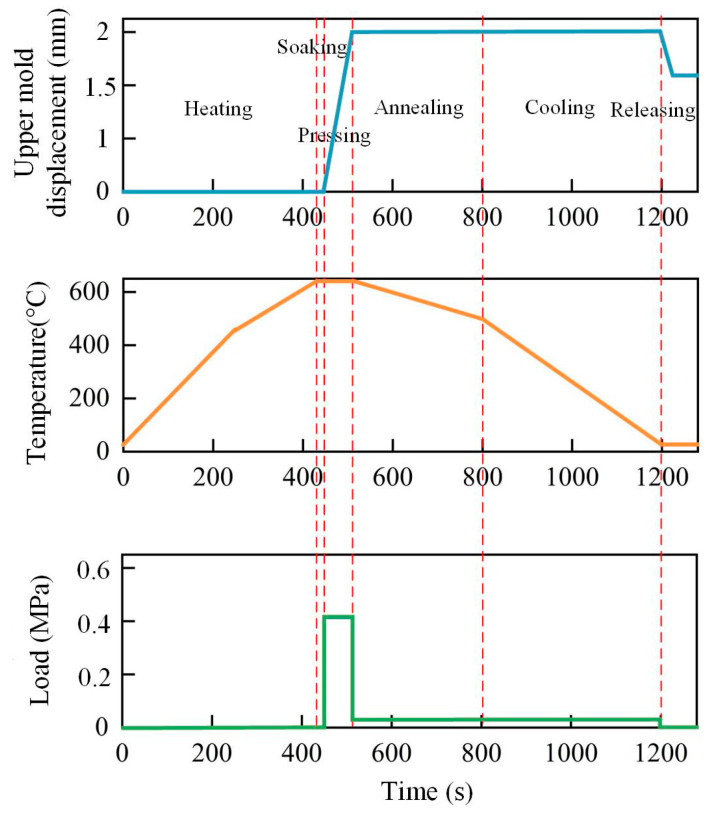
Boundary conditions at different stages of GMP.

The temperature management strategy for the glass-forming process consisted of three main phases: heating, holding, and cooling. The entire heating and soaking process was designed to last approximately 430 s, with a constant heating rate of 1.5 °C/s. During the forming stage, under a cylinder constant pressure of 0.4 MPa (output pressure was 3.14 kN), the upper mold moved 10 mm along the negative z direction, successfully completing the GMP. This stage was expected to take 80 s to ensure the glass achieved the desired shape. Following this, the glass component and the mold entered a slow annealing phase to reduce the temperature to around 500 °C, alleviating internal stress within the glass. Subsequently, the temperature was rapidly dropped to 25 °C to ensure the stability and durability of the glass component. A critical step in this process involved applying a constant force of about 400 N on the upper surface of the mold, which was essential to prevent deformation of the glass component during the annealing and cooling phases, thus maintaining its intended shape and accuracy. Figure 10 illustrates the setting of the boundary conditions. Additionally, in the simulation, a stick–slip friction model was employed to simulate the contact behavior between the glass and the mold. In this model, the thermal conduction coefficient between the mold and glass was set as 2800 W/m^2^K [3,42], the coefficient of friction was set to 0.1, the friction coefficient multiplier was 1.05, the transition zone from sliding friction to viscous friction was set to 10^−6^, and the friction force tolerance was set to 0.05 [29].

**Figure 11:**
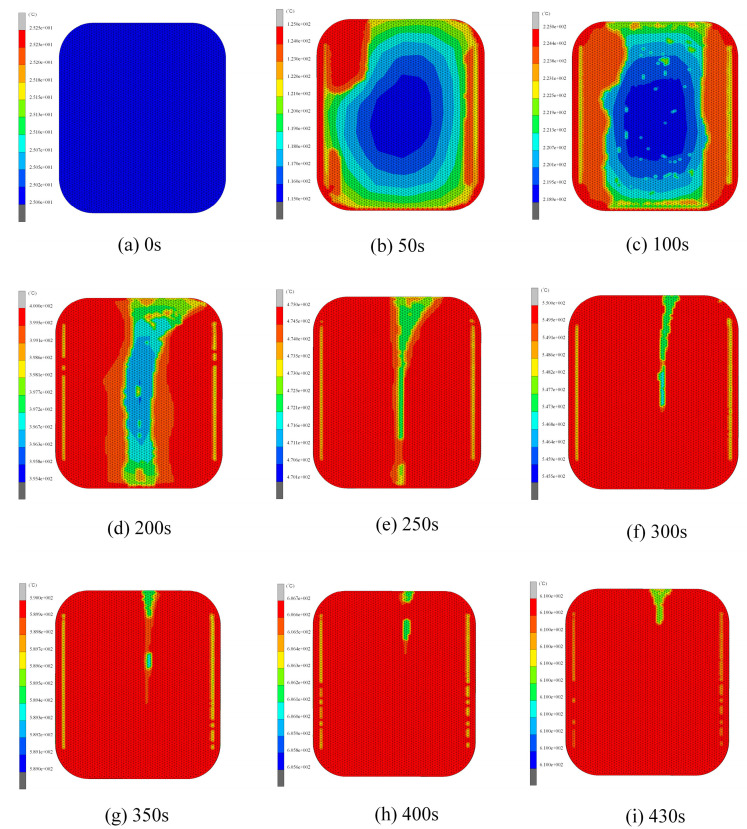
Temperature distribution of the ultra-thin glass component during heating and equilibration phases: (**a**) 0 s, (**b**) 50 s, (**c**) 100 s, (**d**) 200 s, (**e**) 250 s, (**f**) 300 s, (**g**) 300 s, (**h**) 400 s, (**i**) 430 s (setting parameters: heating rate = 1.5 °C/s, holding time = 80 s, forming temperature = 610 °C, forming pressure = 0.4 MPa, cooling rate = 1 °C/s).

The temperature distribution of ultra-thin glass components for smartwatches at various heating stages is illustrated in Figure 11. A dynamic trend is observed, where extended heating times result in varying surface temperatures of the glass component. In the initial heating stage, the surface temperature difference in the glass component was as high as 10 °C (in Figure 11b). This phenomenon was primarily due to contact heat conduction from the bottom mold, which served as the main heat source, causing higher temperatures at the central part of the glass compared with the edges. This temperature variation arose mainly because the mold did not directly contact the edges of the glass. Over the course of the 300 s heating process, as the furnace temperature gradually increased, the glass component’s temperature differential decreased to about 6.1 °C (in Figure 11f), indicating that continued heating results in a more uniform temperature distribution across the glass components. The process then transitions to the soaking stage, during which the temperature reached the required forming temperature of 610 °C (in Figure 11i).

### 4.2. Influence of Molding Process Parameters

The primary factors determining the quality of 3D curved screen glass are the surface quality and dimensional accuracy of the manufactured part. Surface or internal defects in a molded part can arise from various sources, including residual stresses, manufacturing conditions, and surface imperfections. Additionally, energy consumption during the GMP is a significant concern. The forming process parameters have a substantial impact on both the energy consumption and quality of the produced glass components. Therefore, an in-depth analysis of these parameters is essential. Such analysis not only optimizes the production process and reduces energy consumption but also enhances the overall quality of glass components. By examining the influence of forming process parameters on forming quality, more efficient and environmentally friendly glass manufacturing can be achieved, ensuring superior product performance.

In calculating energy consumption, the heat absorbed by the mold, glass, and nitrogen, as well as the heat generated by the use of nitrogen, are considered. Equation (12) represents the sum of the energy consumption of each part of the production process, which may include mold heating energy consumption, glass heating energy consumption, nitrogen heating energy consumption, etc. The total energy consumption of a production cycle can be calculated by the following formula (see Equations (12) and (13)):

(12) $$\;\;E_{e}=\lambda (Q_{1}+Q_{2})=\lambda (\sum_{i=1}^{2}c_{i}m_{i}\Delta T+c_{3}vt\rho \Delta T^{\prime })$$

(13) $$\lambda (T)=2.01+3.1\times 10^{-5}T$$

where

$E_{e}$—power consumption (KJ/pcs);
λ—the heat loss coefficient, which is a function of temperature T;
$Q_{1},Q_{2}$—heat absorbed by mold and glass and consumed by nitrogen gas (KJ/pcs);
$C_{1\sim 3}$—specific heat capacity of mold, glass, and nitrogen (J/(kg °C));
$m_{i}$—quality of molds and glass (kg);
ΔT—temperature changes in molds and glass (°C);
$\Delta T^{\prime }$—nitrogen temperature change (°C);
ν—nitrogen flow rate (mL/s);
t—nitrogen inflow time (s);
ρ—nitrogen density (kg/mm^3^).

#### 4.2.1. Single-Factor Experimental Design

Single-factor experiments are crucial for determining the optimal thermal bending glass molding process parameters. Based on a comprehensive review of the related literature and actual GMP parameters, five key process parameters were identified, and five sets of single-factor experiments were designed for each parameter. This study focused on process parameters such as molding temperature, molding pressure, heating rate, cooling rate, and pulse pressure frequency during the GMP. Table 5 details the parameter values and experimental conditions for these experiments. In the experimental design, scientific methodological principles were followed. For each set of experiments, only one parameter was varied while the other four parameters remained constant. This variable control method ensured the accuracy and comparability of the results, allowing for a precise assessment of the impact of each parameter change on the thermal bending glass forming effect. By comparing and analyzing the results from each set of experiments, the specific influence of each process parameter on the thermal bending glass forming effect was derived. These valuable experimental data provide a critical basis for subsequent multifactor optimization, facilitating the identification of the best combination of process parameters to achieve high-quality and efficient production of glass hot-forming.

#### 4.2.2. Effect of Molding Temperature on Glass Forming Quality

**Figure 12:**
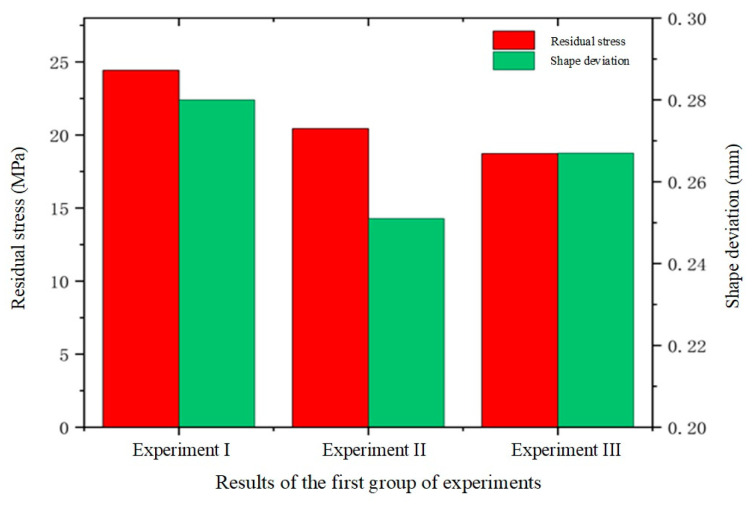
Simulation results of the first set of experiments (molding temperatures of 610 °C, 620 °C, and 630 °C, respectively).

**Figure 13:**
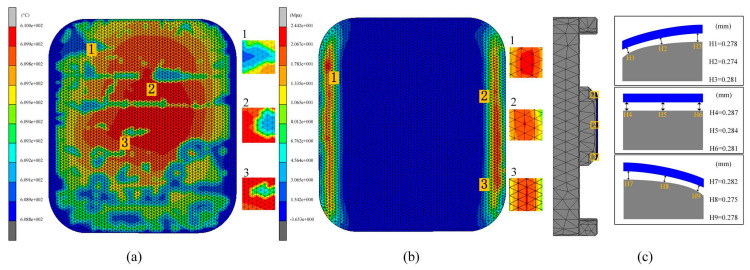
Simulation results of the first set of experiment I (molding temperature 610 °C), (**a**) temperature distribution, (**b**) residual stresses, (**c**) shape deviations.

**Figure 14:**
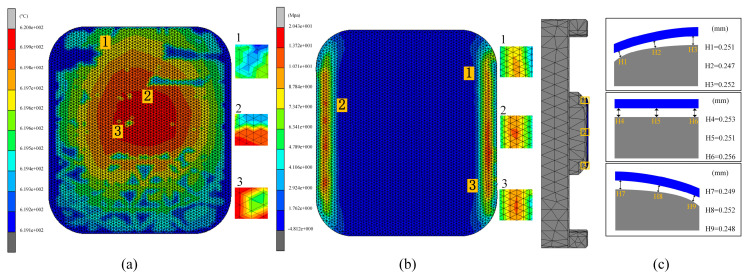
Simulation results of the first set of experiment II (molding temperature 620 °C), (**a**) temperature distribution, (**b**) residual stresses, (**c**) shape deviations.

**Figure 15:**
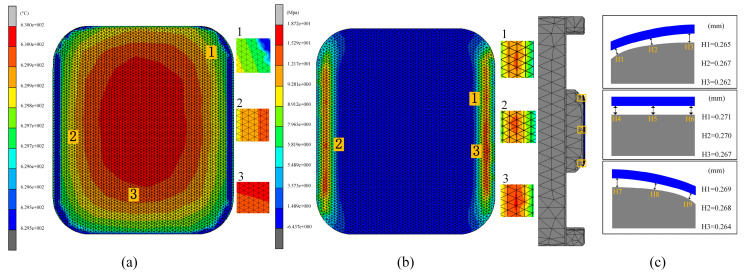
Simulation results of the first set of experiment III (molding temperature 630 °C), (**a**) temperature distribution, (**b**) residual stresses, (**c**) shape deviations.

In the first simulation experiment, the effect of different molding temperatures on glass quality was investigated. In the experiment, three forming temperatures, 610 °C, 620 °C, and 630 °C, were set and other process parameters (heating rate, holding time, molding pressure, cooling rate) were kept constant. The simulation experimental results are shown in Figure 12, Figure 13, Figure 14 and Figure 15. The illustrative drawings with labeled values effectively represent clear shape deviation patterns. These visualizations directly highlight the key areas of deviation, providing immediate insight into the characteristics of the shape deviations.

When the mold temperature was set to 610 °C, the overall temperature distribution of the glass components was observed. As shown in Figure 13a, the maximum temperature difference within the glass assembly reached 1.2 °C during mold forming. Under a mold pressure of 30 MPa, the glass buckled and twisted, generating residual stresses known as internal reversal forces. These stresses are not entirely eliminated during the molding process due to the glass’s mechanical and physical property limitations. The presence of residual stresses poses a potential threat to the functionality of glass components, potentially impairing optical properties and mechanical strength, thus affecting the overall quality and service life of the product. Therefore, proper management and treatment of these internal residual stresses are crucial during production. In experiment I, significant residual stresses and shape changes were observed in glass components at a molding temperature of 610 °C. The maximum residual stresses reached 24.42 MPa, as shown in Figure 13b, primarily concentrated in regions prone to bending. This concentration may be attributed to the greater deformation and temperature gradient experienced in these areas. Additionally, the simulation experimental results indicated an average shape change of 0.280 mm for the glass component, as shown in Figure 13c. It was evident that the fit at the edge of the glass component was lower than in the central region. This was due to the maximum fit gap occurring on both sides of the glass. As the mold cooled, the temperature at its edges decreased faster than in the central region, leading to increased cooling shrinkage in the edge region and thus improving the fit between the ultra-thin glass component and the mold.

By comparing Figure 13a, Figure 14a and Figure 15a, the temperature distribution of the prefabricated parts at different temperatures can be clearly observed. As the forming temperature increased, the maximum temperature differences were 1.2 °C, 0.9 °C, and 0.5 °C, respectively. Notably, the maximum temperature difference in all the simulation results did not exceed 1.5 °C. This indicates that the temperature distributions of the glass components were quite consistent after molding, verifying that the three sets of molding temperature parameters used in this study were reasonable. However, at the end of the cooling phase, residual stresses were found to persist within the glass, indicating that the generation of residual stresses was unavoidable. The maximum residual stresses were determined to be 24.42 MPa, 20.43 MPa, and 18.72 MPa, respectively, by comparing Figure 13b, Figure 14b and Figure 15b. These results clearly reveal an inverse relationship between the forming temperature and the residual stresses in the glass. As the forming temperature increased, the residual stress inside the glass gradually decreased. This finding was important for optimizing the glass forming process to reduce the residual stress level.

The average shape deviations detected for the three different forming temperatures were 0.280 mm, 0.251 mm, and 0.267 mm, respectively, as shown in Figure 13c, Figure 14c and Figure 15c. These results indicate a trend where shape deviation initially decreases and then increases with rising forming temperature. Additionally, in the fabricated 3D ultra-thin glass for smartwatches, the gap between the central position and the lower mold was the largest, while the edge region was closer to the mold. This discrepancy can be attributed to the uneven temperature distribution of the mold during cooling. As the temperature at the mold edges drops faster than at the center, more rapid shrinkage occurs in the edge region. This finding has significant practical implications for guiding future manufacturing processes and optimizing mold design. In summary, these experiments effectively demonstrated the substantial impact of molding temperature on internal residual stresses and the quality of glass forming.

#### 4.2.3. Effect of Molding Pressure on Glass Forming Quality

**Figure 16:**
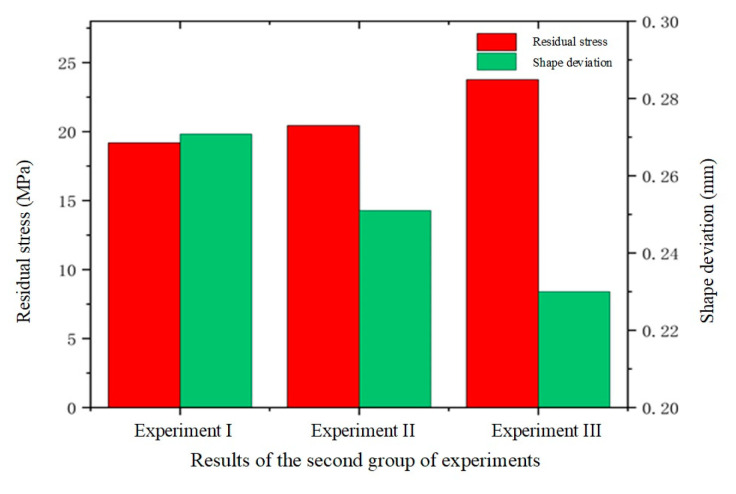
Simulation results of the second set of experiments (molding pressures of 25 MPa, 30 MPa, and 35 MPa, respectively).

**Figure 17:**
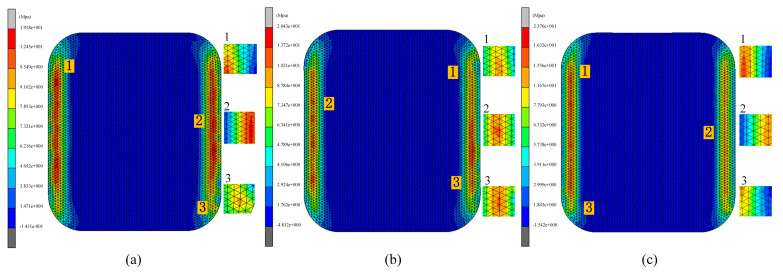
Maximum residual stresses from the simulation results of the second set of experiments (molding pressures of (**a**) 25 MPa, (**b**) 30 MPa, and (**c**) 35 MPa, respectively).

**Figure 18:**
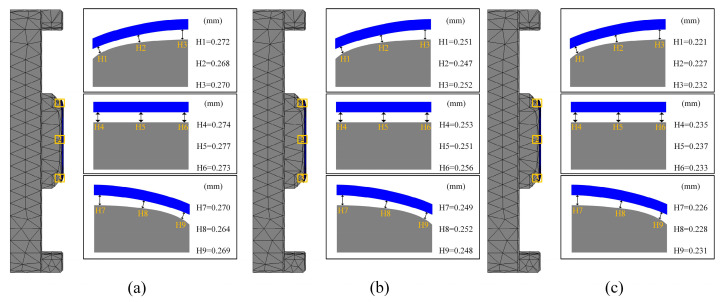
Shape deviation of simulation results of the second set of experiments (molding pressures of (**a**) 25 MPa, (**b**) 30 MPa, and (**c**) 35 MPa, respectively).

In the second set of experiments, the impact of varying molding pressures on glass forming quality was investigated. To gain a comprehensive understanding of this effect, three different molding pressures, 25 MPa, 30 MPa, and 35 MPa, were applied in the simulation experiments of the thermal bending forming process. The results are presented in Figure 16, Figure 17 and Figure 18.

Figure 16 and Figure 17 illustrate that the residual stresses in the glass components increase as the molding pressure rises. The highest residual stress value rose from 19.18 MPa to 23.76 MPa when the molding pressure was increased from 25 MPa to 35 MPa. This issue is linked to an internal material imbalance caused by the glass’s shorter deformation period under increased molding pressure. As internal stress release decreased, the maximum residual stress value correspondingly increased. This indicates that molding pressure significantly affects the generation of residual stresses during the glass forming process.

Figure 16 and Figure 18 reveal a distinct trend in the average shape deviation of the glass assemblies with increasing molding pressure. The average shape deviations as reported by the experimental data were 0.2708 mm, 0.251 mm, and 0.23 mm, in that order. This demonstrates how shape deviation tends to decrease as molding pressure rises. This effect is attributed to the enhanced squeezing force exerted by the mold on the glass as the pressure increases. During the molding process, higher molding pressure improves the fluidity of the glass, facilitating better filling of the mold. The increased fluidity helps to reduce shape deviation, thereby enhancing the overall quality of the molding.

#### 4.2.4. Effect of Heating Rate on Glass Forming Quality

**Figure 19:**
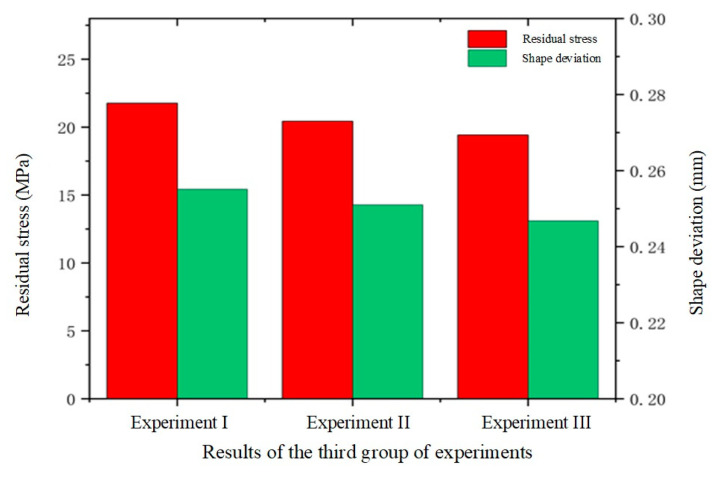
Simulation results of the third set of experiments (heating rates of 1.0 °C/s, 1.5 °C/s, and 2.0 °C/s, respectively).

**Figure 20:**
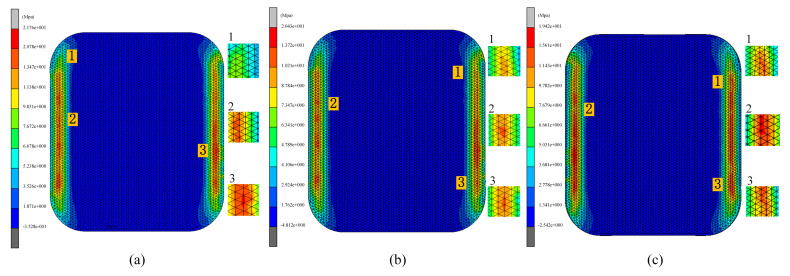
Maximum residual stresses from the simulation results of the third set of experiments (heating rates of (**a**) 1.0 °C/s, (**b**) 1.5 °C/s, and (**c**) 2.0 °C/s, respectively).

**Figure 21:**
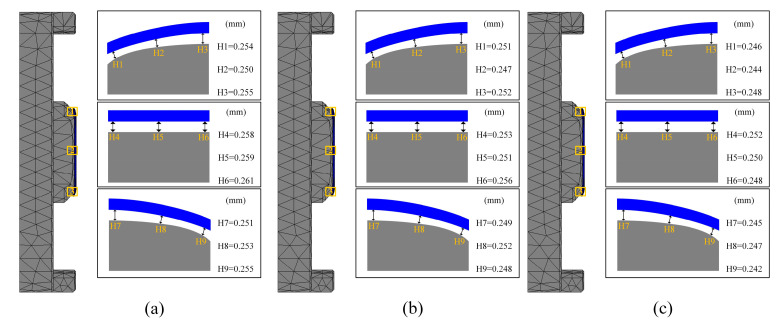
Shape deviation of the simulation results of the third group of experiments (heating rates of (**a**) 1.0 °C/s, (**b**) 1.5 °C/s, and (**c**) 2.0 °C/s, respectively).

In the third set of experiments, the effect of different heating rates on the quality of glass forming was emphasized. In order to fully understand this effect, three different heating rates of 1.0 °C/s, 1.5 °C/s, and 2.0 °C/s were set and simulation experiments of the thermal bending process were conducted. The simulation experimental results are shown in Figure 19, Figure 20 and Figure 21.

Figure 19 and Figure 20 illustrate the relationship between residual stress and heating rate in glass components. The data reveal that residual stresses decrease as the heating rate increases, indicating an inverse relationship between the two. Specifically, the maximum residual stress in the glass component decreased from 21.76 MPa to 19.42 MPa as the heating rate rose from 1.0 °C/s to 2.0 °C/s. This effect was attributed to the accelerated change in the molecular structure of the glass material at higher heating rates, which reduced residual stresses throughout the forming process.

Figure 21 also illustrates the effect of heating rate on shape deviation during the heating phase. As the heating rate increases, shape deviation decreases, attributable to the improved rheological properties of the glass at higher heating rates, which enhance its ability to conform to the mold shape, thereby reducing deviation. Specifically, as the heating rate increased from 1.0 °C/s to 2.0 °C/s, the average shape deviations were observed to be 0.2551 mm, 0.251 mm, and 0.2468 mm, respectively.

#### 4.2.5. Effect of Cooling Rate on Glass Forming Quality

**Figure 22:**
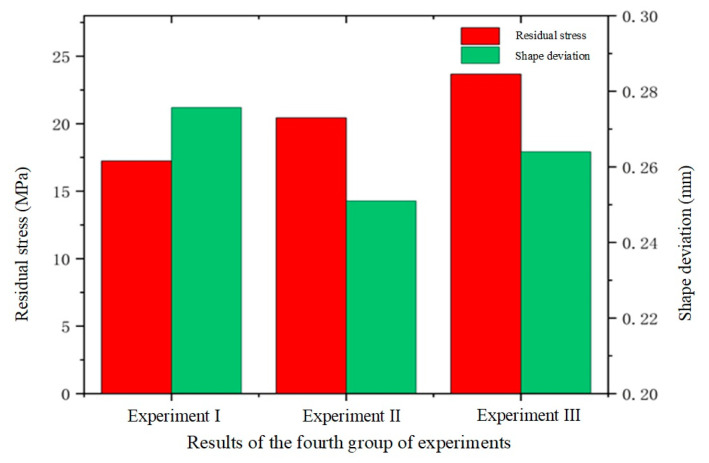
Simulation results of the fourth set of experiments (cooling rates of 0.5 °C/s, 0.75 °C/s, and 1.0 °C/s, respectively).

**Figure 23:**
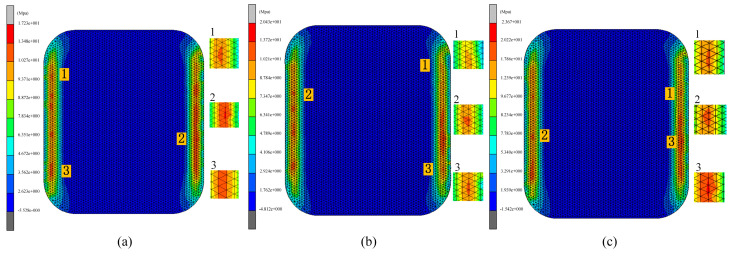
Maximum residual stresses from simulation results of the fourth set of experiments (cooling rates of (**a**) 0.5 °C/s, (**b**) 0.75 °C/s, and (**c**) 1.0 °C/s, respectively).

**Figure 24:**
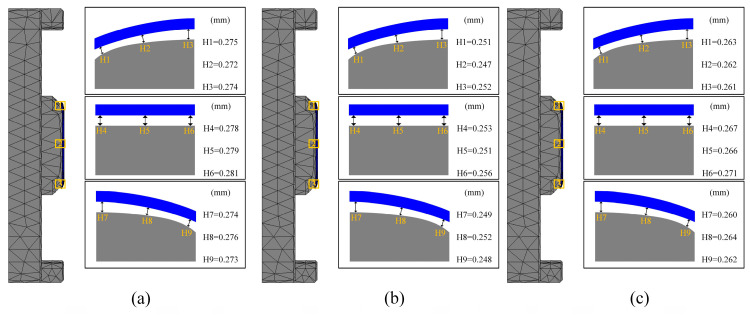
Shape deviation of simulation results for the fourth set of experiments (cooling rates of (**a**) 0.5 °C/s, (**b**) 0.75 °C/s, and (**c**) 1.0 °C/s, respectively).

Cooling rate is a critical parameter in the glass forming process. Excessively rapid cooling can induce significant temperature gradients within the glass, potentially leading to residual stresses or thermal cracks, while excessively slow cooling can extend the production cycle and decrease efficiency. Therefore, selecting an appropriate cooling rate is essential for ensuring high-quality glass forming. In the fourth set of experiments, the effects of various cooling rates on glass forming quality were investigated. The thermal bending process was simulated at cooling rates of 0.5 °C/s, 0.75 °C/s, and 1.0 °C/s. The simulation results are presented in Figure 22, Figure 23 and Figure 24.

Figure 22 and Figure 23 illustrate a positive correlation between residual stress and cooling rate. The glass component’s residual stress increased significantly with increasing cooling rates, reaching 17.23 MPa, 20.43 MPa, and 23.67 MPa at 0.5 °C/s, 0.75 °C/s, and 1.0 °C/s, respectively. Faster cooling rates caused rapid cooling of the glass surface while the internal temperature remained relatively high, leading to increased temperature gradients and consequently higher residual stresses. In contrast, lower cooling rates reduced the volume change in the glass by minimizing the temperature difference between the internal and external layers, thereby decreasing residual stresses.

The relationship between cooling rate and shape deviation in the glass assembly is a little more nuanced, though. Shape deviation first rises with the cooling rate but then falls, as shown in Figure 22 and Figure 24. Specifically, the average shape deviations were 0.2757 mm, 0.251 mm, and 0.264 mm for cooling rates of 0.5 °C/s, 0.75 °C/s, and 1.0 °C/s, respectively. This trend may be attributed to the initial increase in shape deviation due to high cooling rates, followed by a reduction as structural relaxation properties of the glass began to mitigate the deviation. However, this pattern is not universally applicable, and cooling rates may need to be adjusted according to specific conditions to optimize forming quality in practical production settings.

#### 4.2.6. Effect of Pulse Pressure on Glass Forming Quality

**Figure 25:**
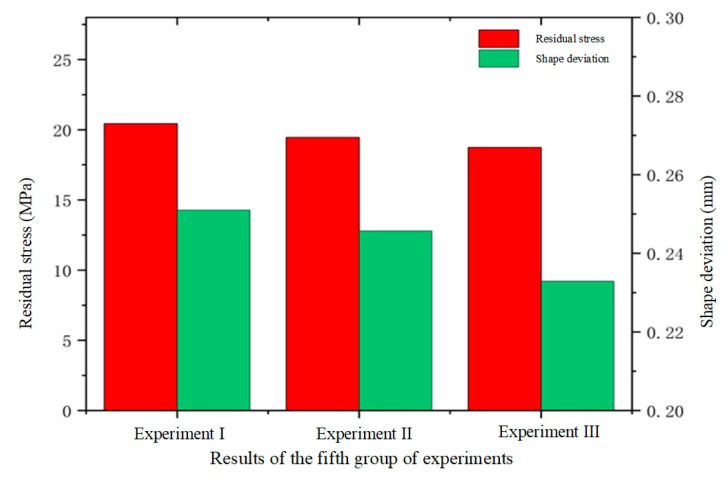
Simulation results of the fifth set of experiments (pressure frequencies of 0 Hz, 30 Hz, and 50 Hz, respectively).

**Figure 26:**
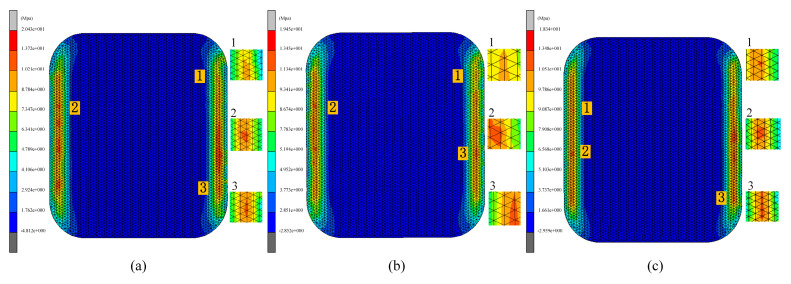
Maximum residual stress from simulation results of the fifth group of experiments (pressure frequencies of (**a**) 0 Hz, (**b**) 30 Hz, and (**c**) 50 Hz, respectively).

**Figure 27:**
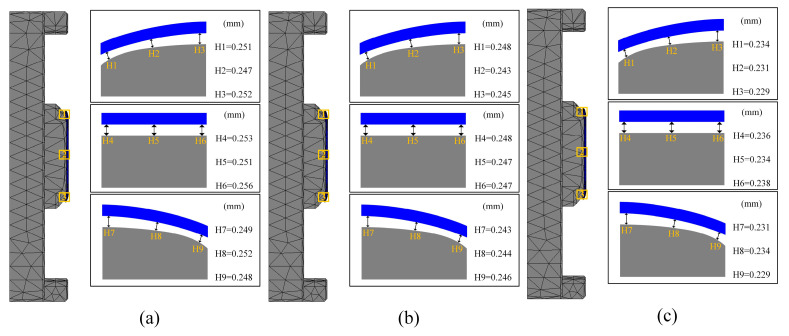
Shape deviation of the simulation results of the fifth group of experiments (pressure frequencies of (**a**) 0 Hz, (**b**) 30 Hz, and (**c**) 50 Hz, respectively).

This subsection examines the impact of pulse pressure on glass forming quality in the fifth set of experiments. Pulse pressure refers to the cyclic variation of pressure applied to the material during the thermal bending process at a specific frequency. Different pulse pressure frequencies were used in the simulation, where the frequency indicated the rate of pressure changes, or the number of pressure variations per second. Pulse pressure frequencies of 0 Hz, 30 Hz, and 50 Hz were simulated during the thermal bending process. The results of these simulations are presented in Figure 25, Figure 26 and Figure 27.

As shown in Figure 25 and Figure 26, the residual stresses in the glass members tended to decrease with the increase in the pulse pressure frequency, which indicated that there was an inverse relationship between them and the frequency. The simulation experimental results show that the residual stresses gradually decreased with the increase in the frequency of pulsation pressure. This may be attributed to the fact that increasing the frequency of pulsation pressure helped to promote faster rearrangement of the molecular structure of the glass material, thus reducing the accumulation of internal stresses during the formation process. Specifically, the residual stress data of 20.43 Mpa, 19.45 Mpa, and 18.74 Mpa for the three sets of experiments clearly demonstrated this trend. At the same time, the increase in pulse pressure frequency also helped to eliminate the inhomogeneity and inconsistency in the forming process by rapidly changing the shape of the glass. When the frequency was increased, the pulse pressure adjusted the shape of the glass material more effectively, resulting in a more uniform and consistent final formed glass with reduced shape deviation. As shown in Figure 25 and Figure 27, the average shape deviation data of the three sets of experiments were 0.251 mm, 0.2457 mm, and 0.2329 mm, respectively.

## 5. Experimental Validation

**Figure 28:**
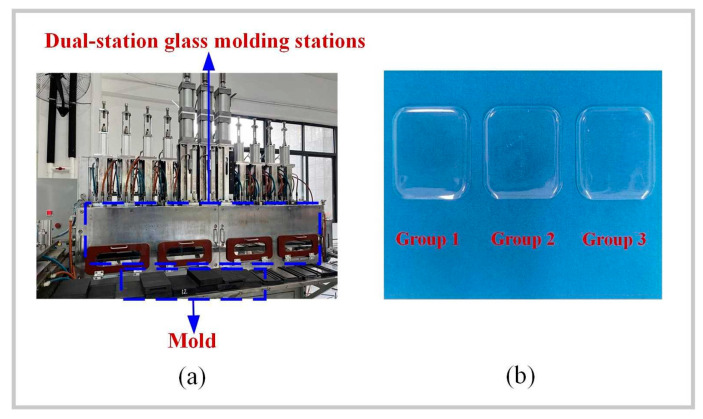
Three-dimensional ultra-thin glass thermal bending machine: (**a**) heating systems, (**b**) experimental.

The glass component molding experiments in this study were conducted using a dual-station molding machine, manufactured by the Intelligent Robotics Institute of Guangdong Province, as shown in Figure 28a. This equipment features a dual-station design, allowing for simultaneous processing tasks at two stations within one cycle, significantly enhancing production efficiency. Typically, a dual-station molding machine comprises main components such as mold heating systems, glass feeding systems, molding systems, cooling systems, and demolding systems. During operation, raw glass materials were preheated and softened in the heating system before being fed into the molding system for shaping. The molded glass products were then rapidly cooled in the cooling system to maintain shape stability. Finally, the products were removed from the molds using the demolding system, producing finished glass components. The primary advantage of this dual-station design was its ability to perform cooling and demolding operations at one station while conducting molding operations at the other. This alternating operation enabled continuous production, significantly boosting equipment productivity and efficiency.

To validate the effectiveness of single-factor simulations of shape deviation (*S_d_*) in glass components, numerical simulation experiments were conducted for Group 1, Group 2, and Group 3, as shown in Figure 28b. By comparing the experiment results with the selected single-factor experimental outcomes, a more precise evaluation of the simulation’s accuracy could be achieved. Using the measurement function in the MSC. Marc 2020 simulation software, the geometric dimensions and shape data of the simulation model were obtained. During the experimental phase, precise measurements of the glass workpiece were taken using a coordinate measuring machine (CMM) to collect the same geometric feature data. The measurement points on the workpiece were aligned with those on the simulation model. A set of reference points was selected to align and transform the coordinate systems, ensuring that both sets of data could be compared within the same coordinate system. The absolute error between the simulated results and the actual measurements at each corresponding measurement point were calculated to assess the shape deviation error. The data points obtained from the CMM served as the basis for analyzing the shape deviation by comparing the measured points with the simulation results reference points. This method provides accurate information on localized deviations, which aligns with the focus of our analysis. Regarding the values of Sd in Table 6, they were obtained by calculating the deviation of the measured point deviations relative to the simulation results’ points. Each point measured by the CMM was compared to its corresponding point on the theoretical design, and the deviation values were used to compute the standard deviation Sd. Table 6 illustrates the relative errors in shape deviation for Groups 1, 2, and 3 between the simulated and experimental results: 8.3%, 5.2%, and 10.6%, respectively. Overall, the experimental results align well with the simulation data, with all relative errors remaining below 20%, indicating the simulation’s effectiveness. The flaws found in traditional simulation modeling are thought to be common and difficult to remove because of the intrinsic constraints of simulation software [43,44]. Research in this area is essential given the high accuracy and miniaturization required for smart wristwatch or touchpad components [45]. To achieve both quality and performance, very specific molding procedures are required. According to this study, the relative error range based on the simulation model was about 0.2 mm, whereas the actual manufacturing shape deviation range for smartwatch glass components was roughly ±0.15 mm. With the possible variables in the experiment and the intrinsic constraints of simulation modeling, it is considered appropriate for this research to keep the relative error level below 20%.

## 6. Conclusions

This study utilized G-11 glass for the simulation analysis and employed both a heat transfer model and a thermal bending simulation model to simulate the glass components of smartwatches. The simulation results were then analyzed and experimentally validated. A summary of the principal findings is provided below, in the following:

(1) The numerical analysis of the heat transfer simulation results underscores the significant impact of the heating rate strategy on energy efficiency. By optimizing with heating strategy 4, which applied an initial heating rate of 35 mJ/(mm^2^·s) during the initial phase (0 to 60 s) and subsequently increased to 45 mJ/(mm^2^·s) during the second phase (60 to 160 s), the system’s thermal output was reduced by 4.396%, while the heating time was shortened by 7.875%.
(2) To study the GMP parameters, a simulation model for the molding of watch glass components was created. By comparing the numerical simulation results, it was found that within the temperature range of 615–625 °C, a molding pressure of 25–35 MPa, a heating rate of 1.5–2.5 °C/s, a cooling rate of 0.5–1 °C/s, and a pulse pressure of 45–55 Hz, the influence on residual stress, and shape deviation in the glass, was minimal.
(3) The results of the thermal bending simulation were validated through experimentation, and the process parameters were analyzed. The simulated findings and the experimental validation were rather close; the maximum deviation did not rise above 20%, thus it was still within a reasonable range.

## Figures and Tables

**Table 1:** Thermal and mechanical properties of WC, SUS 310S, and graphite materials.

Properties	WC	SUS310S	Graphite
Young’s modulus *E* (MPa)	5.7 × 10^5^	1.93 × 10^5^	1.02 × 10^4^
Poisson rate *ν*	0.22	0.3	0.25
Density *ρ* (g/cm^3^)	14.65	7.9	1.78
Thermal conductivity *K* (W/m·°C)	63	18.5	151
Specific heat *C*_p_ (J/kg·°C)	314	500	720
Thermal expansion coefficient (/°C)	4.9 × 10^−6^	18.2 × 10^−6^	4.8 × 10^−6^

**Table 2:** Initial boundary conditions of the simulation model.

Model	Displacement Constraints	Load (MPa)	Initial Temperature (°C)
Upper heating plate	x/y	0.4	800
Upper heat conduction plate	x/y	**-**	760
Mold	x/y	**-**	30
Lower heat conduction plate	x/y/z	**-**	770
Lower heating plate	x/y/z	**-**	810

**Table 3:** G-11 glass and graphite materials’ mechanical and thermal characteristics.

Properties	Glass	Graphite
Young’s modulus *E* (MPa)	7.26 × 10^4^	1.02 × 10^4^
Poisson ratio *ν*	0.206	0.25
Density *ρ* (g/cm^3^)	2.51	1.78
Thermal conductivity *K* (W/m·°C)	1.1	151
Specific heat *C*_p_ (J/kg·°C)	858	720
Thermal expansion coefficient (/°C)	Liquid 3.43 × 10^−5^ Solid 1.143 × 10^−5^	4.8 × 10^−6^

**Table 4:** Parameters of stress relaxation and structural relaxation in G-11 glass.

Stress Relaxation		Structural Relaxation	
Shear Modulus (MPa)	Relaxation Times (s)	Weight Coefficient	Relaxation Times (s)
12,566	0.0689	0.108	3.0
		0.443	0.671
12,615	0.0065	0.166	0.247
		0.161	0.091
4582	0.0001	0.046	0.033
		0.077	0.008

**Table 5:** Control factors and their standard settings.

Experimental Group Number	Experiment No.	Controlled Factors				
		Molding Temperature *A* (°C)	Molding Pressure *B* (MPa)	Heating Rate *C* (°C/s)	Cooling Rate *D* (°C/s)	Pulse Pressure Frequency *E* (Hz)
Group 1	I	610	30	1.5	0.75	0
	II	620	30	1.5	0.75	0
	III	630	30	1.5	0.75	0
Group 2	I	620	25	1.5	0.75	0
	II	620	30	1.5	0.75	0
	III	620	35	1.5	0.75	0
Group 3	I	620	30	1.0	0.75	0
	II	620	30	1.5	0.75	0
	III	620	30	2.0	0.75	0
Group 4	I	620	30	1.5	0.5	0
	II	620	30	1.5	0.75	0
	III	620	30	1.5	1.0	0
Group 5	I	620	30	1.5	0.75	0
	II	620	30	1.5	0.75	30
	III	620	30	1.5	0.75	50

**Table 6:** The results of the experiment and the relative error.

Group	Control Factors					Experimental Results	Relative Error
	*A*	*B*	*C*	*D*	*E*	*S_d_* (mm)	*S_d_* (%)
1	620	25	1.5	0.75	0	0.2932	8.3
2	620	30	2.0	0.75	0	0.2596	5.2
3	620	30	1.5	0.75	0	0.2651	10.6

## Data Availability

The original contributions presented in the study are included in the article, further inquiries can be directed to the corresponding author.
